# Silk fibroin hydrogels from the Colombian silkworm *Bombyx mori* L: Evaluation of physicochemical properties

**DOI:** 10.1371/journal.pone.0213303

**Published:** 2019-03-04

**Authors:** Augusto Zuluaga-Vélez, Diego Fernando Cómbita-Merchán, Robison Buitrago-Sierra, Juan Felipe Santa, Enrique Aguilar-Fernández, Juan C. Sepúlveda-Arias

**Affiliations:** 1 Grupo Infección e Inmunidad, Facultad de Ciencias de la Salud, Universidad Tecnológica de Pereira, Pereira, Colombia; 2 Facultad de Ingenierías, Instituto Tecnológico Metropolitano—ITM, Medellín, Colombia; 3 Grupo de Tribología y Superficies, Universidad Nacional de Colombia, Sede Medellín, Colombia; Fondazione Istituto Italiano di Tecnologia, ITALY

## Abstract

Hydrogel scaffolds are important materials in tissue engineering, and their characterization is essential to determine potential biomedical applications according to their mechanical and structural behavior. In this work, silk fibroin hydrogels were synthesized by two different methods (vortex and sonication), and agarose hydrogels were also obtained for comparison purposes. Samples were characterized by scanning electron microscopy, infrared analysis, thermo-gravimetrical analysis, confined compression test, and rheological test. The results indicate that nanofibers can be obtained via both silk fibroin and agarose hydrogels. The mechanical tests showed that the Young’s modulus is similar to those found in the literature, with the highest value for agarose hydrogels. All the hydrogels showed a shear-thinning behavior. Additionally, the MTT test revealed that silk fibroin hydrogels had low cytotoxicity in THP-1 and HEK-293 cells, whereas the agarose hydrogels showed high toxicity for the THP-1 cell line. The results indicate that silk fibroin hydrogels obtained from a Colombian silkworm hybrid are suitable for the development of scaffolds, with potential applications in tissue engineering.

## Introduction

A general strategy used in tissue engineering is to replace damaged tissue with polymeric scaffolds containing specialized populations of viable cells [1]. The scaffolds may have different forms and incorporate signals or growth factors to stimulate the expansion of a desirable cellular population. Once the platform is implanted, the polymeric scaffold must degrade so it can be replaced by healthy and functional tissue [2]. Hydrogels are useful materials for tissue regeneration due to their compatibility with bioactive agents such as cells and proteins [3]. They also have the ability to transport substances by diffusion to reach physiological concentrations similar to those of the target tissue [4].

Hydrogels are cross-linked networks of polymers undissolved in a water matrix, and they can be used as scaffolds in tissue engineering. Moreover, they have applications as biosensors, vehicles for controlled release of substances, and fill material in soft tissue surgery, among other [5]. Hydrogels may be based on synthetic and/or natural polymers; however, natural polymers are advantageous for tissue engineering because of their biocompatibility and biodegradability. However, because of the characteristics of some tissues such as articular cartilage, bone and intervertebral disc, the resistance of the material is a key factor, and therefore its use is limited by its ability to withstand various mechanical stressors [6]. Accordingly, several authors [7–10] have studied the mechanical properties of hydrogels, but there is no agreement between the reported values and the methods used to determine these properties. Among the natural polymers, agarose and silk fibroin stand out due to their proven characteristics such as low immunogenicity, high permeability to oxygen and water, easy processing, and high stability [11,12].

Agarose is a polysaccharide extracted from red algae of the genera *Gelidium* and *Gracilaria* and consists of repetitions of agarobiose units (L- and D-galactose) [13]. This material has been used to encapsulate molecules [14] and as a scaffold in tissue engineering, due to its controlled degradation, and its ability to maintain the cellular phenotype and to emulate different tissues such as cartilage, bone, skin and cornea [15].

On the other hand, silk fibroin is a fibrillar protein extracted from the cocoons of some arthropods such as the silkworm *Bombyx mori* L. The basic unit of silk fibroin consists of disulfide-linked heavy and light chains and a p25 glycoprotein in molar ratios of 6:6:1 resulting in a complex of approximately 2.3 MDa [16]. The heavy chain has alanine and glycine as its main components, resulting in a water-insoluble chain. Silk fibroin is easily extracted through degumming, and it can be processed to obtain biodegradable and biocompatible materials [17].

Silk fibroin hydrogels can be produced using several methods, such as vortexing, sonication, or application of an electrical current [18]. Different processing methods change the physicochemical properties of the hydrogel. However, there are no studies available in the literature describing the differences in the physicochemical properties between silk fibroin and agarose hydrogels using different processing methods. Therefore, the aim of this work was to obtain silk fibroin hydrogels by sonication and vortex methods, using cocoons from a Colombian silkworm hybrid. Hybridization of silkworm (crossing more than two parental breeds) has been used to produce more resistant breeding to different environmental conditions [19]. In Colombia, hybridization was applied by combining Japanese and Chinese breeds to obtain a new hybrid (Pilamo 2) with a double crossing of (K30 X K05) X (CLS X CHS). The hybridization caused an increase in the cocoon weight and the amount of raw silk. Pilamo 2 is the only silkworm currently marketed in Colombia [20]. In this work, the silk fibroin obtained from the hybrid silkworm was used to prepare hydrogels. The silk fibroin hydrogels were characterized and its potential for biomedical applications was also evaluated. In addition, agarose was used as reference material for this purpose.

## Materials and methods

### Materials and reagents

Colombian hybrid *Bombyx mori* L. silkworm cocoons were collected at the experimental farm El Pílamo at the Universidad Tecnológica de Pereira. All reagents used for cell culture, including Dulbecco's Modified Eagle's Media (DMEM), RPMI Medium 1640, fetal bovine serum (FBS), phosphate-buffered saline (PBS), antibiotics, and trypsin, were purchased from Life Technologies, while agarose was acquired from Fisher BioReagents, Inc. All solutions were prepared with ultrapure water (UPW) generated by a Millipore Direct-Q 8 purification system.

### Fabrication of silk fibroin hydrogels

Silk fibroin solutions were prepared using the process described by Rockwood *et al*. [18] with some modifications. Ten grams of cocoons were cut into small pieces and degummed by boiling in an aqueous solution of 0.02 M Na_2_CO_3_ for 30 min. After rinsing four times using UPW at room temperature, the silk fibroin was dried in an oven at 40°C for 8 hours. The dry silk fibroin was solubilized at 20% (w/w) in a 9.3 M aqueous LiBr solution at 60°C for 24 hours. This solution was dialyzed with ultrapure water for 48 hours using 6,000–8,000 MWCO regenerated cellulose dialysis tubing (Fisherbrand).

Subsequently, the solutions were autoclaved for 15 minutes at 121°C and centrifuged twice at 10,000 rpm for 25 minutes to remove formed silk fibroin aggregates. The final silk fibroin solutions used to obtain the hydrogels were stored (maximum one week) at 4°C in order to avoid aging of silk fibroin. The final silk fibroin concentration was determined by comparing the mass of the solution to the mass of silk after drying at 60°C for 12 hours. For sonication-induced gelation, 5 mL aliquot of 4% (w/v) silk fibroin solution was sonicated with Microson XL 2000. The sonication time was 15 seconds at 20 W. For vortex-induced gelation, 1 mL of 4% (w/v) silk fibroin solution was mixed for 10 minutes at 3,000 rpm using a vortex mixer (Fisher Scientific, Hampton, NH) [21]. All the solutions were incubated at 37°C for a 12 hours period.

### Fabrication of agarose hydrogels

Agarose powder (0.2 g) was added to 10 mL of phosphate-buffered saline (PBS), and the solution was autoclaved for 15 min. Once the agarose cooled to 39°C, it was diluted with PBS to yield a 2% w/v agarose solution, then pipetted into cylindrical molds. After the molds were cooled at 4°C for 10 min, the hydrogels were allowed to equilibrate in PBS for at least 24 h before use [22].

### Samples characterization

#### Surface morphology of hydrogels

The surface morphology of dried hydrogel samples was analyzed via scanning electron microscope (SEM, JEOL 7100F FEG-SEM). The hydrogels were cooled at -20°C for 4 hours and dehydrated by freeze-drying at collector temperature of -50°C for 1 day [23,24]. The obtained dried hydrogels were fractured and coated with gold in order to obtain a conducting material, enabling the morphological study via SEM. The sputtered coatings were obtained in a dual-head Quorum Q300TD sputtering system. Fiber diameter for the dried hydrogels was determined manually from SEM images using the Line tool in ImageJ [25]. Two experienced practitioners independently measured fiber diameters at 25 random locations in each micrograph. A total of 100 data points per sample were analyzed.

#### Infrared spectroscopy analysis

Infrared analysis was done using Fourier transform infrared spectroscopy (FTIR) in attenuated total reflectance (ATR) mode in a Shimadzu IRTracer-100 spectrometer. Samples were analyzed in dry condition, and spectra were acquired with 16 scans at a resolution of 4 cm^−1^ over the 600–4,000 cm^−1^ spectral region.

#### Thermogravimetric analysis (TGA)

Thermal properties were studied by simultaneous TGA-DSC analysis using TA Instruments SDT-Q600 equipment. Samples (10–15 mg) were loaded onto an alumina pan and heated in a nitrogen atmosphere from 25 to 600°C at a heating rate of 5°C/min.

#### Mechanical tests

The mechanical behavior of hydrogels was analyzed using elasticity and viscoelasticity theories. These theories are based on the recovery of the orientation and structure of material as a function of time, although they can also be analyzed independently [7]. The techniques and test equipment used for polymeric materials are also used for the mechanical characterization of hydrogels to be implemented in tissue engineering. Mechanical properties were measured by modifying the method described by Wang *et al*. [26] for unconfined compression tests. Three samples were evaluated for each reported group and tested with an AG-X 100 Shimadzu machine equipped with a 500 N load transducer. Every sample was compressed at an extension-controlled speed of 1 mm/min, starting after the nominal tare loads were reached and sample heights were recorded. The compression stress and strain were determined by normalizing sample geometries, and the traditional elasticity modulus was calculated as the slope of tangent established in stress–strain curves.

#### Rheological tests

Rheological properties were determined using a dynamic torsional shear test. A Haake Mars 40 Rheometer Thermo Scientific with parallel plate geometry was used for the tests. A small compressive strain was applied to maintain contact and prevent slip. Rheological analysis was performed at room temperature with a gap size of 3 mm.

### MTT assay

MTT has been widely used in the literature to evaluate cytotoxicity and cell proliferation in hydrogels [27,28]. In this work, MTT assay was selected because it was readily available. In addition, some considerations were taken into account to be able to detect significant changes in the absorbance at 570 nm without interferences such as the use of 24-well plates to increase the number of cells in the assay and the use of media without phenol red. HEK-293 cells were seeded at 3 x 10^4^ cells/mL, and THP-1 cells were seeded at 6 x 10^4^ cells/mL in 24-well plates containing the hydrogels. After treatment for 24, 48, 72, 96, and 120 h, 500 μL of MTT (5 mg/mL) were added into a culture media without phenol red, after which, cells were cultured for another 4 h. Thereafter, the 24-well plate was centrifuged at 1,500 RPM for 5 min, the media with MTT were aspirated, and 500 μL of DMSO were added into each well. Finally, the samples were read at 570 on a microplate reader (BioTek ELx800). To obtain normalized absorbance values, a reference wavelength of 630 nm was used to subtract background absorbance from signal absorbance (OD_570_—OD_630_)_._ Cells cultured without hydrogels were used as control.

### Statistical analysis

All the experiments were performed with at least three replicates. The software GraphPad Prism 6 (GraphPad Software, San Diego, CA, USA) was used for the statistical analyses. p < 0.05 was considered significant. Data are shown as means and their standard deviations.

## Results

**Fig 1:**
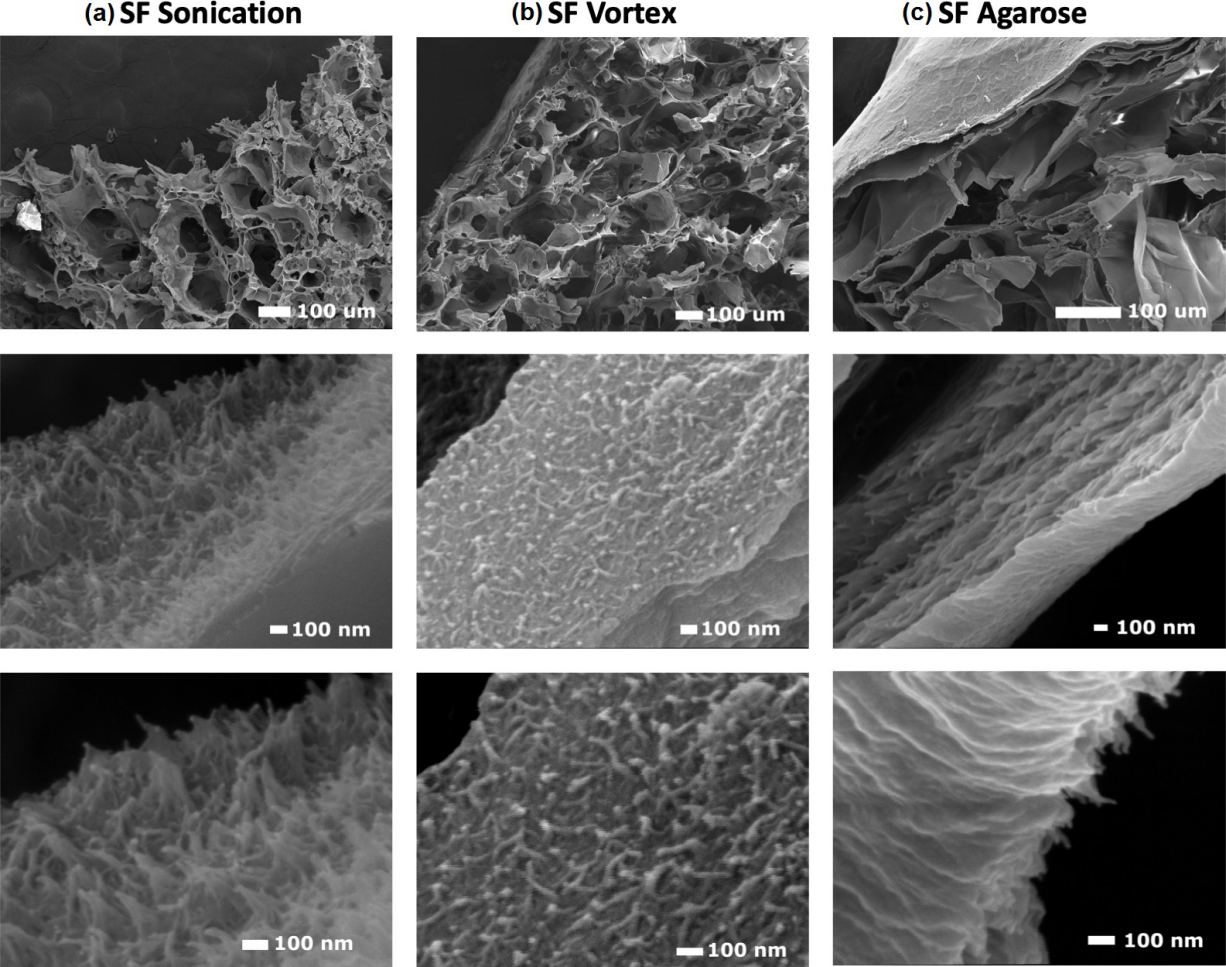
SEM images of (a) silk fibroin hydrogel obtained by sonication method, (b) silk fibroin hydrogel obtained by vortex method, and (c) agarose hydrogel.

Fig 1 shows the SEM images of silk fibroin- and agarose-obtained hydrogels. The diameter of the fibers of the hydrogels was measured at the end of each fiber. Fibers from silk fibroin-based hydrogels had an average diameter of 20 ± 5 nm when they were processed by the vortex method and 21 ± 6 nm by the sonication method. On the other hand, for agarose hydrogels, the fibers had an average diameter of 31 ± 8 nm.

**Fig 2:**
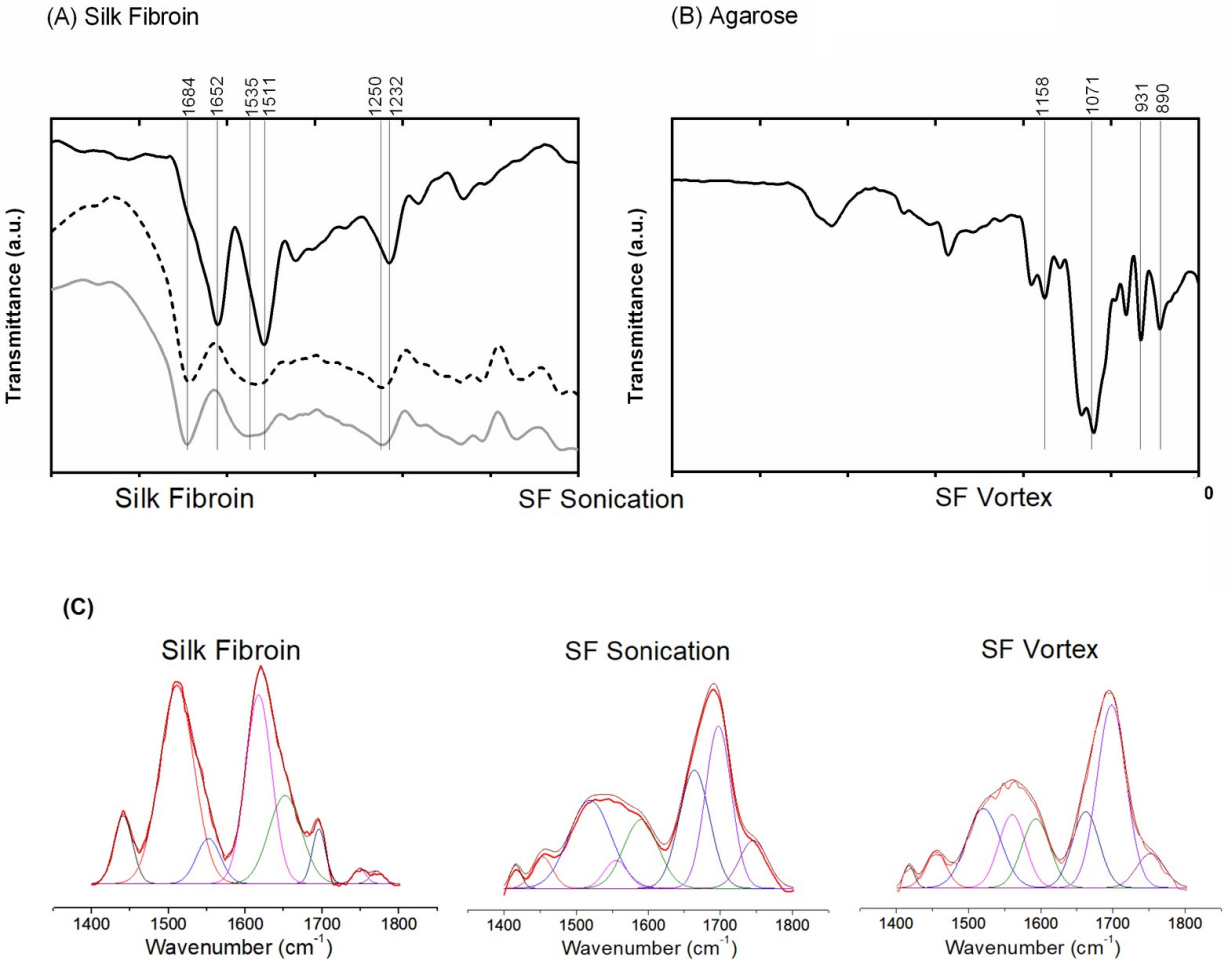
FTIR results for (A) silk fibroin hydrogel from sonication method, vortex method, and silk fibroin solution, (B) agarose hydrogel, (C) Deconvoluted data.

The results of the infrared analysis performed by ATR-FTIR spectroscopy to the hydrogels are shown in Fig 2A. Fig 2B shows the deconvoluted peaks after FTIR analysis (more than 0.999 of correlation was achieved for spectra).The fibroin solution showed peaks of 1,652 cm^-1^ for amide I (C = O vibrations), 1,511 cm^-1^ for amide II (N-H vibrations), and 1,232 cm^-1^ for amide III (C-N vibrations) of silk I secondary structure [29,30], as shown in Fig 2A. In general, higher contents of Amide I bands were found after the gel process using vortex compared with sonication and the silk fibroin solution. The vortex induced gelation during ten minutes allowed intra- and intermolecular interactions of the fibroin chains leading to the formation of β-sheet-rich silk hydrogels [21]. According to Matsumoto et al, the shift of amide I from 1650 to 1625 cm^-1^ is attributable to the β-sheet structure of silk fibroin after gelation [29], however, we did find a displacement of amide I to higher wavenumbers which might be related with the loss of bound water [31,32] induced by the processing of the sample during the FITR analysis (samples were analyzed under dry conditions). Silk fibroin hydrogels (sonication and vortex) exhibited peaks at 1,682–1,686 cm^-1^ for amide I, attributed to Gly-rich amorphous segments or turn structures that allow the formation of intramolecular antiparallel β-sheets [33], 1,531–1,541 cm^-1^ for amide II (secondary N/H bending); and 1,250 cm^-1^ for amide III [34]. The results of the deconvolution are summarized in Table 1.

**Table 1:** Results of deconvoluted area of hidrogels.

Material	Deconvoluted área (%)	
	Amide II (1500–1600 cm^-1^)	Amide I (1600–1700 cm^-1^)
**Silk fibroin**	66	34
**SF Sonication**	57	43
**SF Vortex**	17	83

For the agarose (Fig 2B), FTIR analysis showed the characteristic absorption peaks. Fig 2B shows that the absorption bands of 3,6-anydro-β-galactose and the C-H bending vibrations appeared at 931 and 890 cm^-1^, respectively. A peak related to C-O stretching also appears at 1,073 cm^-1^ [35] as well as peaks at 1,158 and 1,071 cm^-1^ corresponding to -C-O-C- and glycosidic linkage, respectively [36].

**Fig 3:**
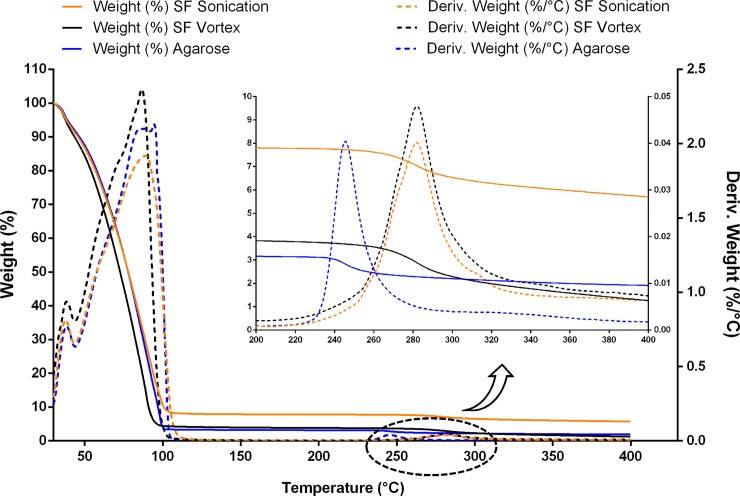
TGA and DTGA thermal analysis of hydrogels.

From TGA and DTGA thermal analysis (Fig 3), three different thermal events can be observed: initial loss of external water near 45°C, major weight loss at 80°C, and degradation temperature at around 260°C. The initial loss of water near 45°C is likely due to the presence of an excess of water outside of the hydrogel structure, and the major weight loss at 80°C is related to the release of bonded water inside the hydrogel [37]. The detail at the top right of Fig 3 shows the thermal degradation for both materials. In the third thermal event, the maximum local degradation rate was found at 247°C for the agarose and at a higher temperature of 282°C for the fibroin. The values found for the fibroin are higher than those found for agarose, and they are in agreement with previously reported values [267°C [38] and 276°C [37], respectively]. From the TGA results, there are no significant differences between the two different gelation methods for the fibroin hydrogels, suggesting similar structural changes during the β-sheet conformation [39].

**Fig 4:**
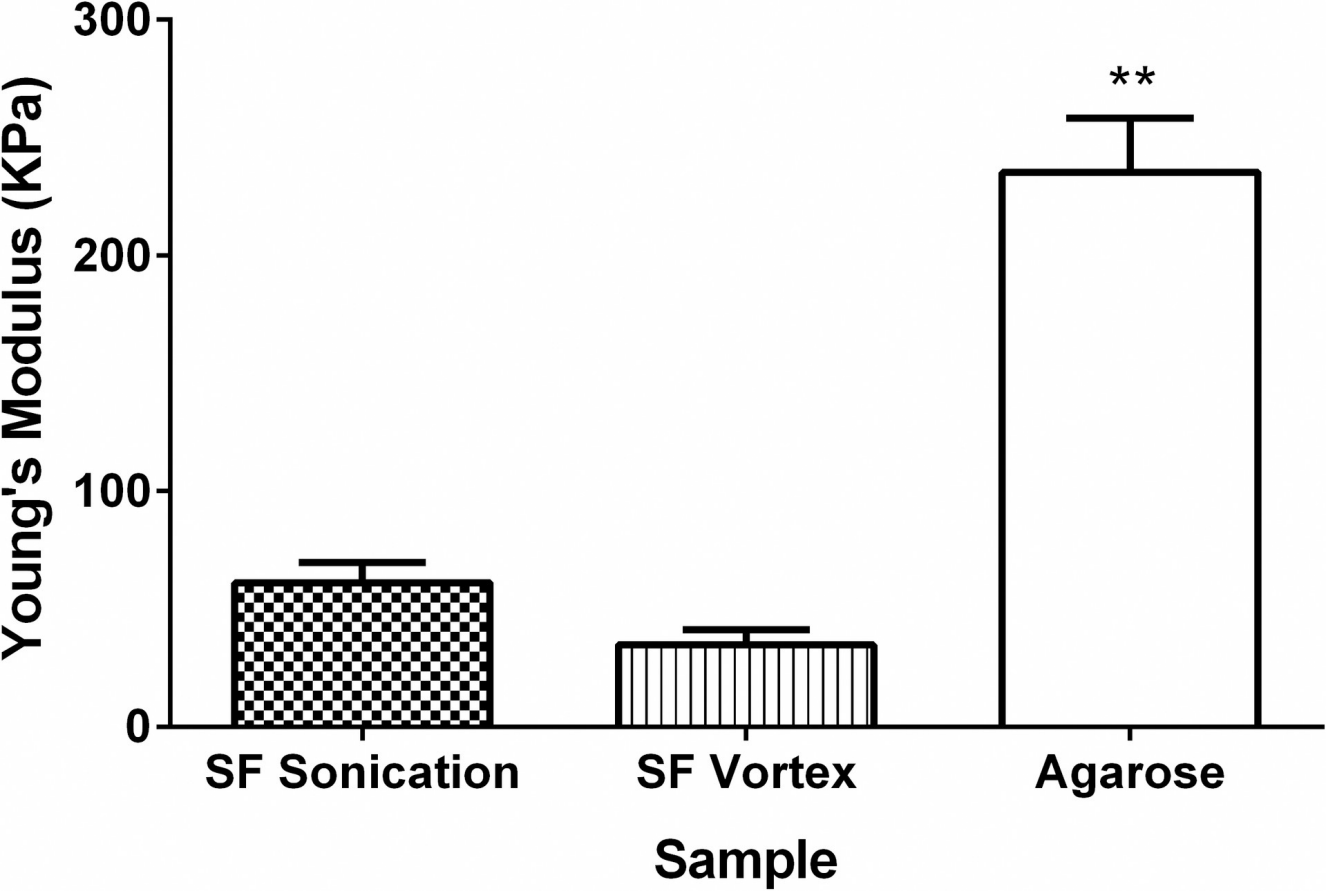
Young’s modulus of samples after mechanical testing. Error bars indicate standard deviations of triplicate measurements. Kruskal-Wallis test. **p<0.01.

Fig 4 shows the Young’s modulus determined from the slope of the tangent in stress–strain curves in an unconfined compression test. The results show that the highest Young’s modulus was found for agarose samples and the lowest value was obtained for vortex samples. Since the results did not follow a normal distribution, Kruskal-Wallis nonparametric analysis was performed. It was possible to establish that the agarose hydrogel was significantly more resistant to compression than the silk fibroin-based hydrogels (**, p < 0.01).

The relationship between shear stress and shear rate in the hydrogels was studied using the Ostwald-de Waele equation [40] (Table 2). All hydrogels showed a non-Newtonian nature characterized by a shear-thinning behavior. Other authors have also found shear-thinning of hydrogels [21], and they stated that this could be attributed to the rupture of dangling chain entanglement crosslinks and rupture of clusters from the hydrogel network.

**Table 2:** Rheological analysis of hydrogels.

Sample	*n*-1	log_10_(*k*)	*n*	*k*	R^2^
Agarose	-0.8015	2.7708	0.1985	589.9293	0.9995
SF Vortex	-0.9424	3.1143	0.0576	1301.0680	0.9920
SF Sonication	-0.9088	3.3106	0.0912	2044.5607	0.9966

*k* = consistency (Pa.s^n^); *n* = power law index; R^2^ = correlation coefficient.

When the data from steady shear are plotted, the measurements for silk hydrogels are described by a power law. This equation expresses apparent viscosity as a function of shear rate for the power law region of a flow curve:

ɳ = k∙γ^(n-1)^

Where *k* = consistency (Pa.s^n^); *n* = power law index; *ɳ* = apparent viscosity (Pa.s) and *γ* = shear rate (s^-1^). At a given temperature, the power law index *n* and the consistency *k* are constants, since they are properties of the fluid. The value of *n* can be determined using a log-log plot of *ɳ* versus *γ*.

For Newtonian fluids, *n* = 1. If *n* < 1, the fluid shows pseudo-plastic (shear thinning) behavior, and for *n* > 1, the flow behavior corresponds to dilatant (shear thickening) fluids. All the hydrogels showed a pseudo-plastic behavior, with very similar results for the silk fibroin hydrogels, with *n* = 0.0576 for vortex hydrogel and *n* = 0.0912 for sonicated hydrogel. For shear rate values under 0.76 s^-1^, the agarose had *n* = 0.1985, indicating a lesser pseudo-plastic character than fibroin hydrogels, followed by a region with a higher *n* value, which is probably a transition region before reaching a Newtonian region at higher values of *γ*.

**Fig 5:**
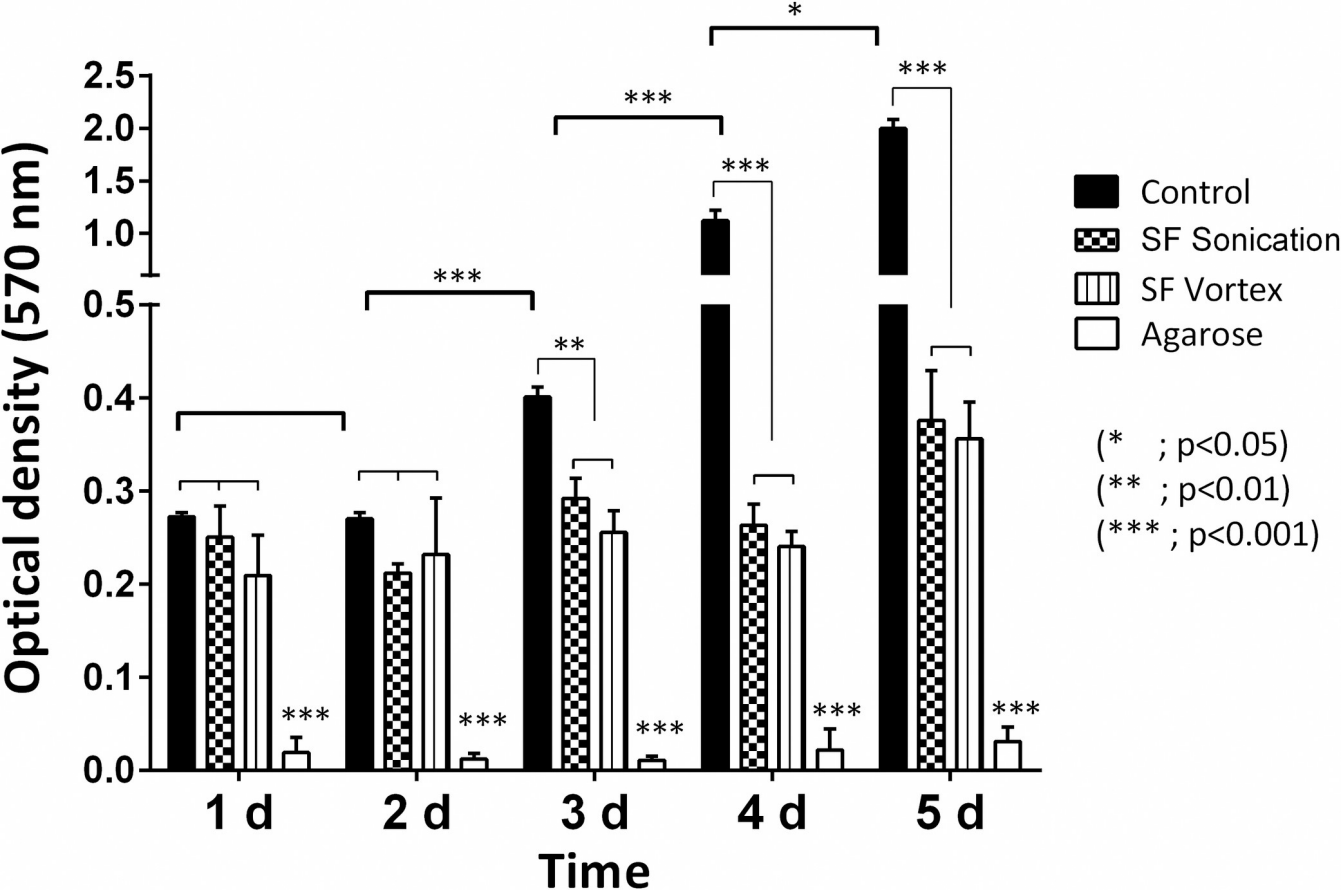
Influence of hydrogels on HEK-293 cell density after an incubation period of 5 days, as determined by the MTT assay. Error bars indicate standard deviations of quadruplicate measurements. Data were analyzed using two-way ANOVA followed by a post-hoc Tukey test.

**Fig 6:**
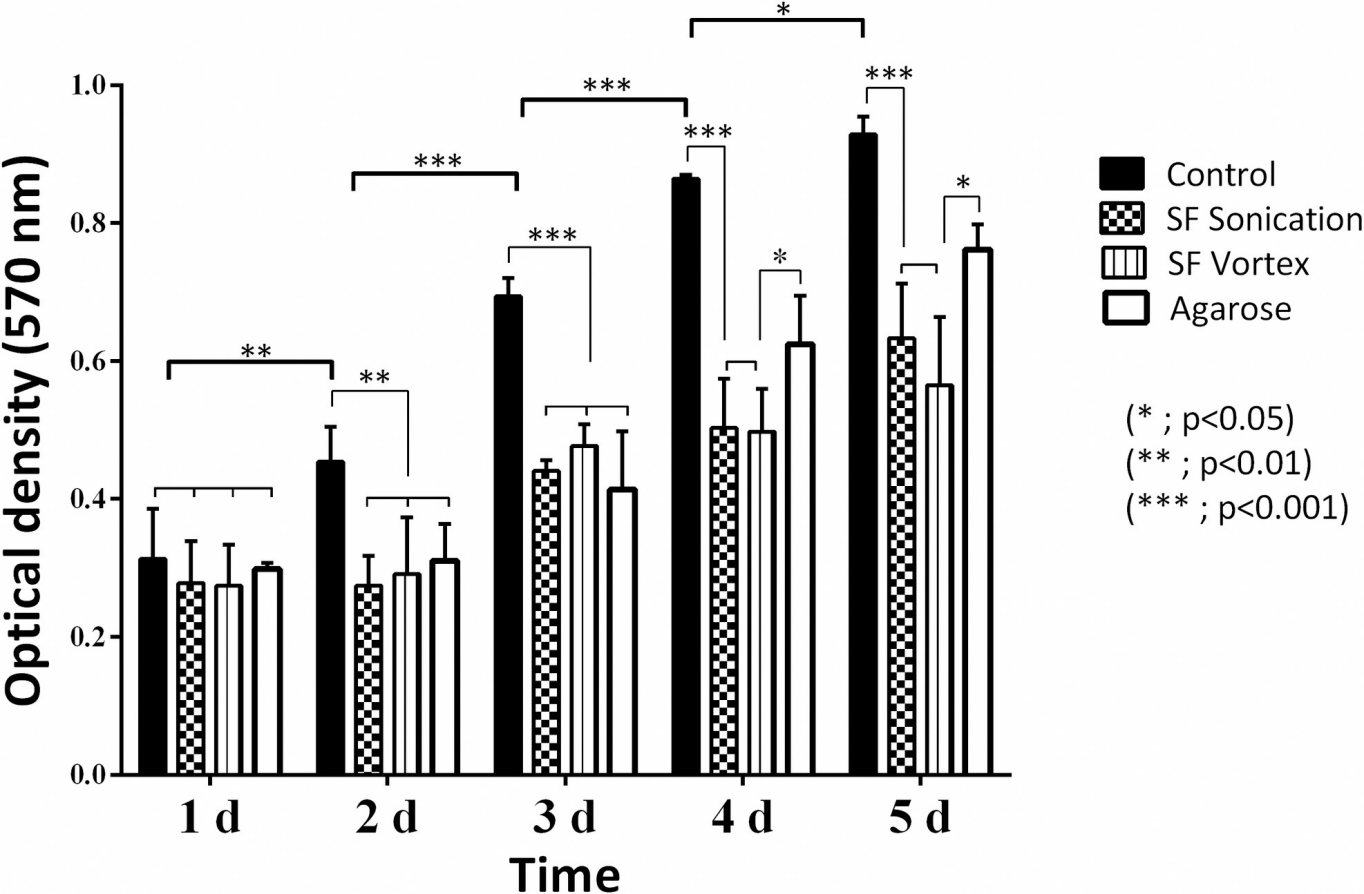
Influence of hydrogels on THP-1 cell density after an incubation period of 5 days, as determined by the MTT assay. Error bars indicate standard deviations of quadruplicate measurements. Data were analyzed using two-way ANOVA followed by a post-hoc Tukey test.

The results of the MTT assay of hydrogels are shown in Figs 5 and 6. Results were compared by a two-way ANOVA, followed by a post-hoc Tukey test. Both types of silk fibroin hydrogels exhibited no differences in terms of cytotoxicity in the cell lines tested. It is clear that both sonication and vortex methods could be used to generate fibroin materials with low toxicity, although the hydrogels showed a decrease in cell proliferation relative to control. The agarose had a high cytotoxicity in adherent cells (HEK-293), but not in the THP-1 cell line. This behavior could be because the latter material does not contain moieties associated with cellular adhesion and adsorption of cell adhesive proteins [41].

## Discussion

Hydrogels are composed of cross-linked nanostructured fibers. The values of the diameters of the hydrogels’ fibers were in agreement with some reports available in the literature, where the diameters of the fibers vary from 3 to 30 nm depending on the experimental conditions [42,43]. Some authors have proposed the use of weak electric fields as a mechanism for the induction of nanofilaments, with similar values of fiber diameters, between 10 and 80 nm [44]. Hydrogels made by annealing using a modulated water/methanol ratio were reported to have nanofibers 10–20 nm in diameter [45]. Besides, the formation of silk fibroin hydrogels by self-assembled nanofiber networks produced fibers with larger diameters (253.2 ± 34.2 nm) [46].

From SEM images, it was found that hydrogels are composed of cross-linked nanostructured fibers, probably due to a mechanical action or a thermal process for the silk fibroin and agarose, respectively. Accordingly, gelation is expected to happen by agglomeration of the polymer-rich domains [47]. From the images, three morphological characteristics can be identified: fiber diameter, layer formation, and entanglement. Therefore, it can be established that the method of silk fibroin hydrogel formation is critical to generate a desired dispersion of the polymer network and appropriate fiber sizes [48]. In all cases, fibers with diameters in the nanoscale are preferred, since their surface area to volume ratio can improve mechanical performance [49], adhesion, proliferation, migration, and cell differentiation [50]. All those properties are desired in tissue engineering applications. High surface area may cause better capture of growth factors and nutrients for the cells. Also, high surface area is important to obtain controlled delivery of drugs through the hydrogels and in tissue engineering for avascular tissues such as cartilage, the epithelial layer of the skin, and the cornea.

The results showed that the silk fibroin is resistant to the steam sterilization process since there was no appreciable effect in fiber morphology and there were no significant changes in the degradation temperature. This result is in agreement with the study developed by Kaplan and workers [51], where they found that sterilization time of 15 minutes is enough to avoid the degradation of silk fibroin or sponges. This finding is valuable since it allows to establish that silk fibroin hydrogels from a Colombian hybrid can be manufactured under aseptic conditions and without the generation of potentially cytotoxic degradation products.

Young's modulus data for the silk fibroin-based hydrogels studied in this work did not match the published results of other researchers (369–1,712 KPa) [26]. This suggests that the material presents a smaller cross-linking associated with a loss of molecular mass that can occur when the degumming treatment exceeds 30 minutes [52]. In addition, the variation of the values found in the literature is significant, and there is no agreement on the method used to measure the mechanical properties among researchers, as seen in Table 3. The initial purpose of this table was to compare the results with those available in the literature. However, when the literature was reviewed it was not possible to directly compare the results since every author measures the mechanical properties using different procedures and techniques such as strain-to failure tests, unconfined and confined compression tests or oscillatory shear measurements. The variation of Young’s modulus found by other authors using both the same materials and techniques is significant. For instance, Kaplan et al. [26] and Tan et al. [9] found variations of one order of magnitude for the same property. Therefore, mechanical properties of these biomaterials are also very sensitive to processing parameters such as silk gel concentration and amplitude during gelation, among others. Then, a comparison with other hydrogels found in the literature was not possible since the measurement techniques and processing parameters differ between articles. In addition, the differences in reported values confirm that this research area is under development and standardized techniques and similar procedures are needed to obtain reproducible results.

**Table 3:** Methods used in the literature to calculate Young’s modulus.

Author	Material	Method	Young’s modulus
Kaplan et *al*. [26]	Silk fibroin hydrogels	A strain-to-failure test was used. The ‘‘traditional” elastic modulus was calculated as the slope of a tangent established at the 5% strain portion of each stress/strain curve.	369–1,712 KPa
Tan et *al*. [9]	Silk fibroin hydrogels	A strain-to-failure test was used. The compressive modulus was calculated from the tangent slope of the linear elastic region of the rendered stress/strain curve.	6.41–63.98 KPa, depending on the silk concentration
Vunjak-Novakovic et *al*. [8]	Hydrogels reinforced with silk microfibers and agarose hydrogels	An unconfined compression test was used. Samples were subjected to a stress-relaxation test to obtain the equilibrium Young’s modulus.	34.0 to 357.2 KPa
Khademhosseini et *al*. [53]	Hydrogels based on gelatin methacrylate and silk fibroin	A compressive stress–strain test was used. The compressive modulus was determined as the slope of the linear region of the stress/strain curve.	~75 KPa for silk fibroin 2% and gelatin methacrylate 6%
Motta et *al*. [54]	Silk fibroin hydrogels	Oscillatory shear measurements were performed to evaluate the storage and loss modulus of hydrogels.	46.1 KPa
Reis et *al*. [55]	Silk fibroin hydrogels	A static compression test was used. The measurements were carried out on the hydrated hydrogel state at 37°C.	110 KPa

On the other hand, from the physicochemical properties of the analyzed silk fibroin hydrogels, it seems that they are suitable for the application of cartilage replacement due to their similar mechanical performance and biocompatibility. Some authors have found that native hyaline cartilage demonstrates compressive moduli on the order of 300–800 KPa [8]. Other tissues with similar mechanical properties are the nucleus pulposus and the annulus fibrosus of the intervertebral discs since they exhibit elastic modules of 5.4 KPa [56] and 75.8 KPa [57], respectively.

Rheological analysis is an appropriate method to characterize the mechanical properties of the hydrogel, as it is fast and sensitive, requires a small sample amount, and reveals differences in the architectures of the materials [58]. The behavior of the hydrogels as non-Newtonian fluids is caused by non-covalent crosslink interactions between the protein chains. Those interactions are progressively interrupted under the influence of the applied shear [59]. Shear-thinning hydrogels do not need any other substance to initiate crosslinking gelation, and accordingly, they can be inserted into tissues without toxic effects. Injectable scaffolds are of great interest for skin regeneration since they can fill irregularly shaped defects through minimally invasive surgical treatments. Given that the human skin has viscoelastic behavior and reported Youngs modulus of 14 KPa [60], it could be interesting to simulate its elastic properties and its micro-architecture through silk fibroin hydrogels. Also, it is crucial to evaluate the ability of the hydrogel to control the bacterial and fungal colonization, taking into account that the biomaterial is composed of an essential nutrient for the growth of microorganisms. As an alternative, the use of this kind of hydrogels for transdermal drug delivery may offer precise control over drug dosage and release kinetics, since the thinning occurs only at the interface between the hydrogel and the needle wall [61].

Biomaterials play an important role in tissue engineering by serving as 2D or 3D frameworks (commonly referred to as scaffolds, matrices, or constructs) for cellular attachment, proliferation, and growth, leading to new tissue formation [50]. In biomaterials design, it is necessary to characterize their cytotoxicity and biocompatibility. Materials displaying low cytotoxicity may be useful for medical applications such as regenerative medicine, in which cell–scaffold unions are required [62]. The evaluation of adherent and non-adherent cell lines provides a broader view of safe degradation of biomaterials and its capacity to promote cell adhesion. Thereby, human embryonic kidney cells (HEK-293) were chosen since they are easy to grow and particularly adequate for studying the substrates effect on cell attachment and growth [63]. Since they are anchorage-dependent cells and need a surface for their proliferation, they are suitable for the screening of the cytotoxic effect of hydrogels. In contrast, the non-adherent cell line (THP-1) allowed establishing the cytotoxic effect of the soluble material present in the hydrogel.

The behavior obtained in the MTT assay of hydrogels depends on cell mobility and the geometry of the well-plate because the cells in the scaffold have a decrease in cellular connectivity [23]. The data suggest that the hydrogel induces cell quiescence and thus may act as a functional niche for some cell populations, such as mesenchymal stem cells (MSCs), which are sought to differentiate into other cell lineages [64].

Agarose does not contain any moieties associated with cellular adhesion and adsorption of cell adhesive proteins [41]. Accordingly, the modification of this material has been discussed with molecules that promote cell adhesion [65] or other conformations of the agarose, such as microspheres, to encapsulate cells [66]. It is important to mention that in THP-1 cells, agarose operated significantly better than silk fibroin when evaluated for prolonged periods, which may be conditioned by the size of the fibers, pore size, and concentration of the polymer in the sample.

The data for the physicochemical properties, mechanical behavior properties, and low cytotoxicity confirm that silk fibroin hydrogels from Colombian hybrid silkworm cocoons have a potential use in biomedical applications.
